# Non-homologous DNA end joining in normal and cancer cells and its dependence on break structures

**DOI:** 10.1590/S1415-47572010005000018

**Published:** 2010-06-01

**Authors:** Tomasz Poplawski, Elzbieta Pastwa, Janusz Blasiak

**Affiliations:** 1 Department of Molecular Genetics, University of Lodz, Banacha, LodzPoland; 2 Department of Molecular Genetics, Medical University of Lodz, Mazowiecka, LodzPoland

**Keywords:** DNA repair, non-homologous DNA end joining, DNA double-strand breaks, complementary and non-complementary DNA ends

## Abstract

DNA double-strand breaks (DSBs) are a serious threat to the cell, for if not or miss-repaired, they can lead to chromosomal aberration, mutation and cancer. DSBs in human cells are repaired *via* non-homologous DNA end joining (NHEJ) and homologous recombination repair pathways. In the former process, the structure of DNA termini plays an important role, as does the genetic constitution of the cells, through being different in normal and pathological cells. In order to investigate the dependence of NHEJ on DSB structure in normal and cancer cells, we used linearized plasmids with various, complementary or non-complementary, single-stranded or blunt DNA termini, as well as whole-cell extract isolated from normal human lymphocytes, chronic myeloid leukemia K562 cells and lung cancer A549 cells. We observed a pronounced variability in the efficacy of NHEJ reaction depending on the type of ends. Plasmids with complementary and blunt termini were more efficiently repaired than the substrate with 3' protruding single-strand ends. The hierarchy of the effectiveness of NHEJ was on average, from the most effective to the least, A549/ normal lymphocytes/ K562. Our results suggest that the genetic constitution of the cells together with the substrate terminal structure may contribute to the efficacy of the NHEJ reaction. This should be taken into account on considering its applicability in cancer chemo- or radiotherapy by pharmacologically modulating NHEJ cellular responses.

The efficient repair of DNA double-strand breaks (DSBs) is essential for the cell to maintain the integrity of its genome. When DSBs are not repaired correctly, they can give rise to chromosomal breakage and translocation. In vertebrate cells, this repair process has evolved in two distinct pathways: homologous recombination repair and non homologous end-joining (NHEJ) (Valerie and Povirk, 2003), the latter relying on Ku (a heterodimer of Ku70 (69 kDa), Ku80 (86 kDa)), DNA-PK_cs_ (DNA protein kinase catalytic subunit), Artemis, Cernunnos, XRCC4 and DNA ligase IV. In the first step of the NHEJ reaction, the heterodimer of Ku70/Ku80 binds to DNA ends. This process activates DNA-PK_cs_ by stabilizing its interaction with DNA termini and recruiting another protein, either Artemis (Less-Miller and Meek, 2003; Lieber *et al.*, 2003) or Cernunnos (Ahnesorg *et al.*, 2006). This complex can trim the DNA ends to make them ligatable. Many other proteins with nuclease and polymerase activities may also contribute to processing DNA ends. Ligation by ligase IV/XRCC4 heterodimer together with Cernunnos is the final step in DSB repair *via* NHEJ. The requirement for DNA ligase IV is imperative, as other DNA ligases are unable to substitute for its function (Chen *et al.*, 2000).

The structure of DNA ends plays an important role in the fast and efficient repair of DSBs by NHEJ (Pastwa *et al.*, 2003; Wang *et al.* 2003). This structure implies, which enzymatic activities will lead the repair process. Complementary and blunt ends can be joined by a simple ligation (Smith *et al.*, 2001). Only small subsets of complementary ends are joined through end processing. Single-strand gaps are filled in by DNA polymerases belonging to the X family - pol μ , pol λ , or terminal deoxytransferases (TdT) (Ma *et al.*, 2004). This mechanism also functions in the processing of non-complementary DNA ends, including 5' protruding single-strand (PSS) / blunt termini, requiring a primer for the fill-in reaction. Priming is provided through the precise alignment of termini by a protein complex (DNA-PK) associated with juxtaposed DNA ends. On the other hand, non-complementary DNA ends can also be joined after small terminal deletion. The core NHEJ enzyme with nuclease activity is Artemis. Other nucleases which can be involved in DSB repair *via* NHEJ are Werner syndrome protein (WRN), an enzyme with nuclease and helicase activity and Mre11 subunit of the Mre11/Rad50/Nbs1 complex (Yannone *et al.*, 2001; D'amours and Jackson, 2002). Mre11 has 3'-to-5' dsDNA exonuclease and ssDNA endonuclease activities. An additional NHEJ processing enzyme is the mammalian polynucleotide kinase (PNK). PNK acts as a 5' -kinase/3' -phosphatase to create 5' -phosphate/3' -hydroxyl termini, which are a crucial requirement for the ligation process. PNK is recruited to the repair complex through interactions with XRCC4 protein (Koch *et al.*, 2004). The ligation of mismatched and non-cohesive DNA ends is facilitated by a recently discovered protein, Cernunnos (Ahnesorg *et al.*, 2006). Cells lacking DNA-PK and DNA ligase IV activity are incapable of efficiently repairing DNA DSBs (Wang *et al.*, 2001). These cells employ another NHEJ pathway, which repairs DNA lesions with 20-30-fold slower kinetics. The slower element of the DNA rejoining system (known as B-NHEJ) is more error-prone and DNA-PK-independent (Wang *et al.*, 2003).

The efficient repair of a whole range of DSBs by NHEJ and B-NHEJ depends on the accessibility of a protein machinery to damaged DNA, which is, at least in part, determined by the structure of DNA termini. Normal and cancer cells have different genetic constitutions and presumably a different ability to repair DSBs by end joining, and they display a dissimilar sensitivity to drugs used in cancer therapy which produce certain kinds of DSBs. To elucidate the molecular mechanisms involved in the processing of various DNA termini *via* NHEJ, we examined the *in vitro* repair of linearized plasmid DNA containing various ends, *viz*., blunt as well as complementary and non-complementary 5' or 3' protruding single-strand (PSS). We used the extracts from normal human lymphocytes, as well as two human cancer cell lines, namely myeloid leukemia K562 and lung epithelial A549. We chose these cell lines because they represented two different types of tumors. K562 cells are derived from blood cancer – they grow very quickly whereas A549 cells represent solid tumors and grow relatively. Both cell lines display different sensitivity to agents used in anticancer therapy – ionizing radiation and topoisomerase II inhibitors, which may be contingent on different DNA repair mechanisms (Long *et al.*, 1985; Chiron *et al.*, 1992; Jeong *et al.*, 2001; Vens *et al.*, 2002). In the A549 cell line, NHEJ protein expression and activities are normal (Long *et al.*, 1985; Diggle *et al.*, 2003; Kasten-Pisula *et al.*, 2007),whereas in K562 cells the expression of such proteins may be down-regulated (Deutsch *et al.*, 2001). Nevertheless, the level of mRNA in non-homologous end-joining genes was high when compared to that in normal human lymphocytes (Chiou *et al.*, 2007). The data obtained may be of aid in increasing chemotherapeutic efficiency with drugs producing specified termini of DNA double-strand breaks.

Peripheral blood lymphocytes (PBL) were obtained from 10 young, healthy, non-smoking donors and isolated by centrifugation in a density gradient of Ficoll (15 min, 230 g). After isolation, the lymphocytes were pooled and their final concentration adjusted to 10^6^ cells/mL by adding the growth medium with phytohemagglutinin (PHA) to the single cell suspension. The human myeloid leukemia cells K562 and lung epithelial cancer cells A549 were maintained in exponential growth in RPMI 1640 supplemented with 10% FBS and 1% streptomycin/penicillin. Cell viability, measured by trypan blue staining, proved to be around 99%.

Cell extracts were prepared as previously described (Pastwa *et al.*, 2005). Briefly, the cells were harvested, washed, pelleted twice in ice-cold PBS (900 *g*), and then resuspended in a hypotonic lysis buffer (10 mM Hepes [4-(2-hydroxyethyl) piperazine-1-ethanesulfonic acid], pH 7.9, 60 mM KCl, 1 mM EDTA pH 8.0, 1 mM DTT) and a protease inhibitor cocktail (according to manufacturer's instructions) (minimum 4-6 10^7^ cells/0.5 mL of extraction buffer). The cells were then lysed by three cycles of freeze-thawing in a bath of dry ice/ethanol and in a 37 °C water bath. We believe that we did not have any DNA, either mitochondrial or nuclear, in our cell-extract, as this was clarified by centrifugation at 15,000 g. The extract was then stored at -70 °C until needed. Protein content was determined by the Bradford assay, using bovine serum albumin as the standard (Bradford, 1976).

Linearized plasmid DNA (pUC19) was prepared by digestion with restriction endonucleases (Fermentas) recognizing a unique site within it. To produce complementary ends, the plasmid was digested with a single restriction enzyme *Hin*dIII. The substrates with non-complementary termini were prepared by means of combinations of two restriction enzymes (*Hin*dIII/*Eco*RI, *Kpn*I/*Pst*I, *Eco*RI/*Pst*I, *Eco*RI/*Hin*cII, and *Sph*I/*Hin*cII) acting separately, and single digestion with *Hin*cII, which produced blunt termini. In the case of double digestion, the reaction with the first enzyme was checked by gel electrophoresis, where upon linearized DNA was precipitated by ethanol and digested with the second. DNA was purified from agarose gel with a DNA extraction kit (Qiagen). Before the end joining assay procedure, a small aliquot of the substrate DNA was subjected to electrophoresis in agarose gel to estimate DNA concentration.

End joining assay was performed as previously described (Pastwa *et al.*, 2001). The repair reactions were carried out in a total volume of 50 μL, containing 50 mM Tris, pH 8.0, 5 mM MgCl_2_, 1 mM ATP, 1 mM DTT, 5% polyethyleneglycol 8000, a protease inhibitor cocktail, 100 ng of substrate DNA and 15 μg of proteins from A549, K562 or lymphocyte whole cell extract. Samples were incubated at 17 °C for 16 h, because we observed the highest efficacy of the NHEJ reaction at this temperature (Pastwa *et al,.* 2001). Repair was stopped by adding 0.4% SDS and incubation at 65 °C for 15 min. DNA was recovered by extraction with phenol:chloroform (1:1 v/v) and ethanol precipitation, using 0.5 μg tRNA as a carrier. Repair products were identified by 1% agarose electrophoresis and staining for 1 h with Vistra Green, according to manufacturer's instructions. For quantification, gels were scanned and bands were analyzed densitometrically using Quantity One 1-D analysis software (Bio-Rad, Hercules, CA, USA).

All the values in this study represent the means ± SD for three separate experiments, each performed in triplicate. One-way analysis of variance was used to evaluate the influence of the structure of DNA ends on the efficacy of DNA repair. The differences between means were compared using Scheffe's multiple comparison test (SPSS Inc., Chicago, IL, USA).

To evaluate whether the ability of human cells to repair DSBs by DNA end joining is affected by the structure of the DNA ends, we used a plasmid-based *in vitro* end joining assay. The plasmid DNA pUC19 has several single restriction sites permitting a creation of double strand breaks with various configurations of complementary and non-complementary termini (Figure 1). In order to estimate end-joining efficacy in whole cell extracts prepared from normal and cancer cells, 100 ng of linearized plasmid DNA was incubated with cellular extract. After 16 h the plasmid DNA was recovered and the end joining reaction products were densitometrically analyzed through agarose gel electrophoresis. End-joining efficiency can be determined as a percentage of multimers (dimers, trimers, etc.) in the original linear DNA substrate. Typical pictures obtained from agarose gel electrophoresis of end joining reaction products are shown in Figure 2. The results are displayed in Figure 3 and Table 1. Ligation efficiency in whole cell extracts prepared from normal and cancer cells were compared. Figure 3 and Table 1 show the ability to join different complementary and non-complementary ends by extracts of human lymphocytes, K562 myeloid leukemia and A549 lung epithelial cancer cells. All kinds of cells were able to repair DNA lesions caused by restriction enzymes, but the efficiency of DNA repair was different and was dependent on the type of cells used for extract production and type of linear DNA ends generated by endonucleases, except for the joining of incompatible 5' overhang DNA substrates. The mean of end joining for all types of investigated DNA ends obtained for extracts of K562 cells was lower by about 30% than that obtained for A549 and human lymphocyte cells. Human lymphocyte extract converted nearly 50% of compatible 5' and 3' linear DNA substrates to linear products (dimers, trimers and high molecular weight products), whereas in the remaining incompatible linear DNA substrates, conversion was about 30%. The highest efficiency of end joining with the A549 whole cell extract was obtained for 3' complementary overhangs. In general all substrates containing 3' overhangs were repaired very efficiently (over mean value) by A549 whole cell extract, except for 3'-blunt substrate. Our results show that the extract from leukemic cells was more efficient in end-joining with substrates containing 5' overhangs than those with 3' ends.

Pronounced variability in NHEJ efficiency, depending on end structure and cell type, was apparent. Only the 5'PSS/blunt termini were repaired with similar efficiency in all cells. In general, single strands were joined with the lowest efficacy. These data are different from results obtained in other laboratories. Smith *et al.* (2001), when using a plasmid-based host-cell end-joining assay demonstrated that repair efficacy of various DNA ends is similar. The difference may result from the fact that we compared end-joining *in vitro*, whereas Smith and colleagues did so *in vivo*. Moreover, SD values of the *in vivo* assay are high, making it therefore difficult to estimate overall efficiency of NHEJ activity in joining DNA ends with various configurations, and then arrive at final conclusions. We showed that repair which does not require end processing, as in the case of complimentary 5' PSSs, 3' PSSs and blunt/blunt, is more effective than that of more complex DNA ends. This is not surprising, since only DNA ligase is involved in the joining of two complementary or blunt DNA ends, thereby making it less energy- and time-consuming than the joining of more complicated DNA termini, generally requiring a multi-protein complex. To evaluate whether the efficacy of repair was influenced by the sequence of the DNA termini, we examined end joining reaction of linearized DNA substrate containing blunt end with GC- and AT-rich sequences, but we did not observe any substantial difference for DNA blunt ends generated by *Hinc*II (GTC|GAC), *Sma*I (CCC|GGG) and *Ssp*I (AAT|ATT) enzymes (data not shown).

We showed that the substrate with 3' PSS non-complementary ends was more efficiently joined by A549 cell extract, in contrast to remaining cell lines. These data suggest that A549 cells may generate mechanisms responsible for a very high efficient repair of DSBs containing 3' PSS non-complementary ends. Repair of these DNA termini *via* NHEJ requires proteins with 3' exonuclease activity. It seems that the A549 cells may be an appropriate candidate for the study of molecular mechanisms of processing of DNA ends in DSB repair *via* NHEJ, due to high effectiveness of this process in these cells.

Our results demonstrate that the overall efficiency of end joining in human leukemia K562 cell extract was lower than in normal human lymphocytes and A549 cells. K562 cells express the BCR/ABL fusion oncogenic tyrosine kinase that causes genomic instability and drug resistance of human leukemia by modulating DNA repair (Blasiak *et al.*, 2002; Majsterek *et al.* 2002; Slupianek *et al.*, 2002;). Furthermore, in contrast to lymphocytes and A549, our K562 cells do not express wild-type p53 tumor suppressor protein, which is involved in DSB repair (Usuda *et al.*, 2003). Our current and previous data suggest that NHEJ seems to be repressed by BCR/ABL in K562 cells (Pastwa *et al.*, 2005). Notably, Deutsch *et al.* (2001) demonstrated down-regulation of the major mammalian DNA repair protein DNA-PK_cs_ by BCR/ABL in BCR/ABL-positive murine and human hematopoietic cells. Another report by van Heemst *et al.* (2004) indicated that blunt ended DSBs are efficiently rejoined in DNA-PK_cs_ deficient cell lines. These findings are in agreement with our results, by demonstrating the highest repair efficiency in whole K562 extract for blunt-ended substrates. Contrary to our results, Gaymes *et al.* (2002) showed that ligation efficiency of 5' PSS complementary ends was increased 2-7 fold in myeloid leukemia cells in comparison to normal peripheral blood lymphocytes. We think that B-NHEJ activity in BCR/ABL-positive leukemia cells is very intense, when compared to normal cells. When DNA-PK dependent NHEJ is lacking, cells must launch B-NHEJ to repair DSBs and survive. In normal cells, B-NHEJ should be silenced by DNA-PK-dependent NHEJ, due to its more error-prone nature and the longer time required in repairing DNA lesions. Therefore, BCR/ABL-positive leukemia cells become genetically unstable by including B-NHEJ in DNA DSB repair. NHEJ activity in BCR/ABL-positive cells differs from that in BCR/ABL-negative, this difference possibly being related to the ratio of DNA-PK-dependent and -independent NHEJ reactions.

It should be noted that similar studies have been carried out with either intramolecular rejoining of re-circularized plasmids (North *et al.* , 1990; Thacker *et al.*, 1992; Ganesh *et al.*, 1993) or intermolecular rejoining of plasmids (Fairman *et al.*, 1992; Derbyshire *et al.*, 1994; Nicolas and Young, 1994; Nicolas *et al.*, 1995). Intramolecular end joining is not a good model for studying NHEJ activity, since only small subset of substrate are rejoined by NHEJ (Ohsaki *et al.*, 2005). All remaining studies used a nuclear extract as a source of NHEJ protein. We assumed that a whole-cell extract would be a better source of NHEJ protein to examine activity of NHEJ in cell free system than a nuclear extract, seeing that NHEJ protein, such as Ku, are present in cytoplasm and are transported to nuclei in the case of DNA double-strand break signaling (Nagasawa *et al.*, 1997). We also used the DNA extraction kit from agarose gel to prepare DNA substrates used in this work. Prepared this way, these substrates are free from contamination by the small DNA fragments which are produced when restriction enzymes cut plasmid vectors. Small DNA fragments and uncut plasmid DNA remaining after digestion of plasmid DNA by two restriction enzymes could interfere in the NHEJ reaction and generate counterfeit end results. Moreover, phenol used in preparation of NHEJ substrates in those studies can damage DNA (Claycamp, 1992). Thus substrates prepared by phenol extraction should not be used in DNA repair assays that evaluate NHEJ activity, as they are a target for BER instead of NHEJ proteins.

Taken together, end-joining efficiency strongly depends on the structure of DNA termini and the nature of the cells. This, of course, is not surprising, but data on the response to DSBs by particular cancer cells may be of assistance when considering the use of drugs and/or irradiation in cancer therapy, by making them more effective for cancer cells and less harmful for normal cells.

**Figure 1:**
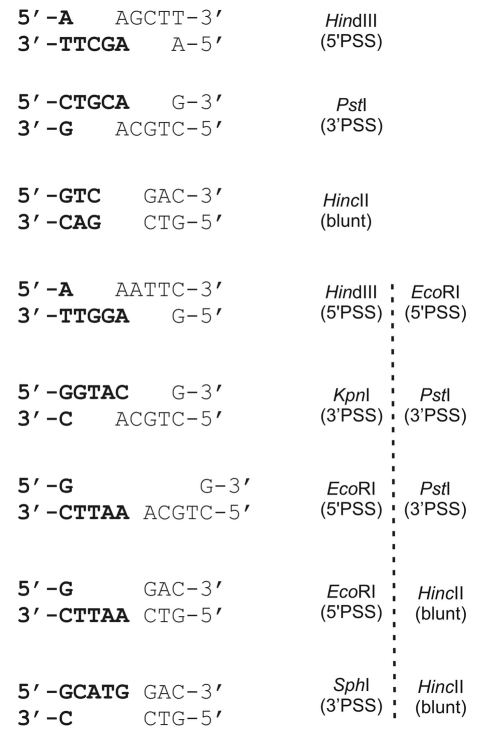
End structures of the DNA substrates used to determine NHEJ efficacy in human cells. The restriction endonucleases used to create the DNA termini are displayed together with the structure of the DNA ends they generated.

**Figure 2:**
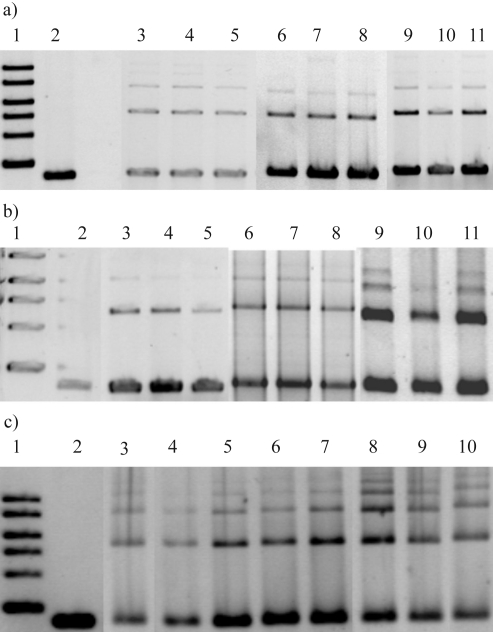
A typical result of agarose gel electrophoresis of the products of end-joining reactions after 16 h incubation of substrate pUC 19 DNA plasmid linearized with *Hin*cII (A), *Eco*RI/*Pst*I (B) and *Pst*I (C), with cell extracts from human lymphocytes, K562 and A549 cells. A - lane 1, 0.5 μg 1 kb DNA; lane 2, *Hin*cII-cut DNA negative control; lanes 3-5, substrate DNA + K562 cell extract; lanes 6-8, substrate DNA + human lymphocyte cell extract; line 9-11, substrate DNA + A549 cell extract. B - lane 1, 0.5 μg 1 kb DNA; lane 2, *Eco*RI/*Pst*I-cut DNA negative control; lanes 3-5, substrate DNA + K562 cell extract; lanes 6-8, substrate DNA + human lymphocyte cell extract; lanes 9-11, substrate DNA + A549 cell extract. C - lane 1, 0.5 μg 1 kb DNA; lane 2, *Pst*I-cut DNA negative control; lanes 3-4, substrate DNA + K562 cell extract; lanes 5-7, substrate DNA + human lymphocyte cell extract; lanes 8-10, substrate DNA + A549 cell extract.

**Figure 3:**
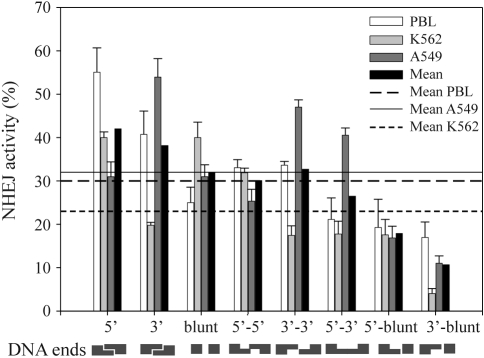
DNA end joining in human lymphocytes, and K562 and A549 cells for different complementary and non-complementary substrates. The structure of analyzed substrate DNAs are schematically displayed below the graph. Horizontal lines represent the mean for each type of cell used in the experiment.

## Figures and Tables

**Table 1:** DNA end joining efficiency in human lymphocytes, K562 and A549 cells.

N.	Enzyme	DNA ends	End joining efficiency [%]		
			Lymphocytes	K562	A549
1	*Hin*dIII	5' PSS/5' PSS	55 ± 5.6	40 ± 1.3	31 ± 3 .4
2	*Pst*I	3' PSS/3' PSS	40 ± 5.3	19 ± 0.6	54 ± 4.2
3	*Hin*cII	blunt/blunt	25 ± 3.8	40 ± 3.5	31 ± 2.7
4	*Hin*dIII/*Eco*RI	5' PSS/5' PSS	33 ± 1.8	31 ± 1.0	25 ± 2.7
5	*Kpn*I/*Pst*I	3' PSS/3' PSS	33 ± 0.8	17 ± 2.2	47 ± 1.6
6	*Eco*RI/*Pst*I	5' PSS/3' PSS	21 ± 4.9	17 ± 2.9	40 ± 1.6
7	*Eco*RI/*Hin*cII	5' PSS/blunt	19 ± 6.1	17 ± 3.5	16 ± 2.6
8	*Sph*I/*Hin*c*II*	3' PSS/blunt	16 ± 3.6	4 ± 1.1	11 ± 1.6

## References

[Ahnesorgetal2006] Ahnesorg P., Smith P., Jackson S.P. (2006). XLF interacts with the XRCC4-DNA ligase IV complex to promote DNA nonhomologous end-joining. Cell.

[Blasiaketal2002] Blasiak J., Gloc E., Mlynarski W., Drzewoski J., Skorski T. (2002). Amifostine differentially modulates DNA damage evoked by idarubicin in normal and leukemic cells. Leukemia Res.

[Bradford1976] Bradford M.M. (1976). A rapid and sensitive method for the quantitation of microgram quantities of protein utilizing the principle of protein-dye binding. Anal Biochem.

[Chenetal2000] Chen L., Trujillo K., Sung P., Tomkinson A.E. (2000). Interactions of the DNA ligase IV-XRCC4 complex with DNA ends and the DNA-dependent protein kinase. J Biol Chem.

[Chiouetal2007] Chiou S.S., Huang J.L., Tsai Y.S., Chen T.F., Lee K.W., Juo S.H., Jong Y.J., Hung C.H., Chang T.T., Lin C.S. (2007). Elevated mRNA transcripts of non-homologous end-joining genes in pediatric acute lymphoblastic leukemia. Leukemia.

[Chironetal1992] Chiron M., Demur C., Pierson V., Jaffrezou J.P., Muller C., Saivin S., Bordier C., Bousquet C., Dastugue N., Laurent G. (1992). Sensitivity of fresh acute myeloid leukemia cells to etoposide: Relationship with cell growth characteristics and DNA single-strand breaks. Blood.

[Claycamp1992] Claycamp H.G. (1992). Phenol sensitization of DNA to subsequent oxidative damage in 8-hydroxyguanine assays. Carcinogenesis.

[DamoursandJackson2002] D'amours D., Jackson S.P. (2002). The Mre11 complex: At the crossroads of DNA repair and checkpoint signaling. Nat Rev Mol Cell Biol.

[Derbyshireetal1994] Derbyshire M.K., Epstein L.H., Young C.S., Munz P.L., Fishel R. (1994). Nonhomologous recombination in human cells. Mol Cell Biol.

[Deutschetal2001] Deutsch E., Dugray A., Karim B.A., Marangoni E., Maggiorella L., Vaganay S., Rasy S.D., M'Kacher R., Eschwege F., Vainchenker W. (2001). BCR/ABL down-regulates the DNA repair protein DNA-PK_cs_. Blood.

[Diggleetal2003] Diggle C.P., Bentley J., Kiltie A.E. (2003). Development of a rapid, small-scale DNA repair assay for use on clinical samples. Nucleic Acids Res.

[Fairmanetal1992] Fairman M.P., Johnson A.P., Thacker J. (1992). Multiple components are involved in the efficient joining of double stranded DNA breaks in human cell extracts. Nucleic Acids Res.

[Ganeshetal1993] Ganesh A., North P., Thacker J. (1993). Repair and misrepair of site-specific DNA double-strand breaks by human cell extracts. Mutat Res.

[Gaymesetal2002] Gaymes T.J., Mufti G.J., Rassool F.V. (2002). Myeloid leukemias have increased activity of the nonhomologous end-joining pathway and concomitant DNA misrepair that is dependent on the Ku70/86 heterodimer. Cancer Res.

[Heemstetal2004] Heemst D., Brugmans L., Verkaik N.S., Gent D.C. (2004). End-joining of blunt DNA double-strand breaks in mammalian fibroblasts is precise and requires DNA-PK and XRCC4. DNA Repair.

[Jeongetal2001] Jeong S.J., Jin Y.H., Moon C.W., Bae H.R., Yoo Y.H., Lee H.S., Lee S.H., Lim Y.J., Lee J.D., Jeong M.H. (2001). Protein tyrosine kinase inhibitors modulate radiosensitivity and radiation-induced apoptosis in K562 cells. Radiat Res.

[Kasten-Pisulaetal2007] Kasten-Pisula U., Windhorst S., Dahm-Daphi J., Mayr G., Dikomey E. (2007). Radiosensitization of tumour cell lines by the polyphenol Gossypol results from depressed double-strand break repair and not from enhanced apoptosis. Radiother Oncol.

[Kochetal2004] Koch C.A., Agyei R., Galicia S., Metalnikov P., O'Donnell P., Starostine A., Weinfeld M., Durocher D. (2004). Xrcc4 physically links DNA end processing by polynucleotide kinase to DNA ligation by DNA ligase IV. EMBO J.

[Less-MillerandMeek2003] Less-Miller S.P., Meek K. (2003). Repair of DNA double strand breaks by non-homologous end joining. Biochimie.

[Lieberetal2003] Lieber M.R., Ma Y., Pannicke U., Schwarz K. (2003). Mechanism and regulation of human non-homologous DNA end joining. Nat Rev Mol Cell Biol.

[Longetal1985] Long B.H., Musial S.T., Brattain M.G. (1985). Single- and double-strand DNA breakage and repair in human lung adenocarcinoma cells exposed to etoposide and teniposide. Cancer Res.

[Maetal2004] Ma Y., Lu H., Tippin B., Goodman M.F., Shimazaki N., Koiwai O., Hsieh C.L., Schwarz K., Lieber M.R. (2004). A biochemically defined system for mammalian nonhomologous DNA end joining. Mol Cell.

[Majstereketal2002] Majsterek I., Blasiak J., Mlynarski W., Hoser G., Skorski T. (2002). Does the BCR/ABL-mediated increase in the efficacy of DNA repair play a role in the drug resistance of cancer cells?. Cell Biol Int.

[Nagasawaetal1997] Nagasawa M., Watanabe F., Suwa A., Yamamoto K., Tsukada K., Teraoka H. (1997). Nuclear translocation of the catalytic component of DNA-dependent protein kinase upon growth stimulation in normal human T lymphocytes. Cell Struct Funct.

[NicolasandYoung1994] Nicolás A.L., Young C.S. (1994). Characterization of DNA end joining in a mammalian cell nuclear extract: Junction formation is accompanied by nucleotide loss, which is limited and uniform but not site specific. Mol Cell Biol.

[Nicolasetal1995] Nicolás A.L., Munz P.L., Young C.S. (1995). A modified single-strand annealing model best explains the joining of DNA double-strand breaks mammalian cells and cell extracts. Nucleic Acids Res.

[Northetal1990] North P., Ganesh A., Thacker J. (1990). The rejoining of double-strand breaks in DNA by human cell extracts. Nucleic Acids Res.

[Ohsakietal2005] Ohsaki A., Iiyama K., Miyagawa Y., Kawaguchi Y., Koga K., Kusakabe T. (2005). Nonhomologous end-joining in a cell-free extract from the cultured silkworm cell line BmN4. Mol Biol Rep.

[Pastwaetal2001] Pastwa E., Neumann R.D., Winters T.A. (2001). *In vitro* repair of complex unligatable oxidatively induced DNA double-strand breaks by human cell extracts. Nucleic Acids Res.

[Pastwaetal2003] Pastwa E., Neumann R.D., Mezhevaya K., Winters T.A. (2003). Repair of radiation-induced DNA double-strand breaks is dependent upon radiation quality and the structural complexity of double-strand breaks. Radiat Res.

[Pastwaetal2005] Pastwa E., Poplawski T., Czechowska A., Malinowski M., Blasiak J. (2005). Non-homologous DNA end joining in normal and leukemic cells depends on the substrate ends. Z Naturforsch.

[Slupianeketal2002] Slupianek A., Hoser G., Majsterek I., Bronisz A., Malecki M., Blasiak J., Fishel R., Skorski T. (2002). Fusion tyrosine kinases induce therapeutic drug resistance by stimulation of homology-dependent recombination repair, prolongation of G2/M phase and protection from apoptosis. Mol Cell Biol.

[Smithetal2001] Smith J., Baldeyron C., De Oliveira I., Sala-Trepat M., Papadopoulo D. (2001). The influence of DNA double-strand break structure on end-joining in human cells. Nucleic Acids Res.

[Thackeretal1992] Thacker J., Chalk J., Ganesh A., North P. (1992). A mechanism for deletion formation in DNA by human cell extracts: The involvement of short sequence repeats. Nucleic Acids Res.

[Usudaetal2003] Usuda J., Inomata M., Fukumoto H., Iwamoto Y., Suzuki T., Kuh H.J., Fukuoka K., Kato H., Saijo N., Nishio K. (2003). Restoration of p53 gene function in 12-*O*-tetradecanoylphorbor 13-acetate-resistant human leukemia K562/TPA cells. Int J Oncol.

[ValerieandPovirk2003] Valerie K., Povirk L.F. (2003). Regulation and mechanisms of mammalian double-strand break repair. Oncogene.

[Vensetal2002] Vens C., Dahmen-Mooren E., Verwijs-Janssen M., Blyweert W., Graversen L., Bartelink H., Begg A.C. (2002). The role of DNA polymerase beta in determining sensitivity to ionizing radiation in human tumor cells. Nucleic Acids Res.

[Wangetal2003] Wang H., Perrault A.R., Takeda Y., Qin W., Wang H., Iliakis G. (2003). Biochemical evidence for Ku-independent backup pathways of NHEJ. Nucleic Acids Res.

[Wangetal2001] Wang H., Zeng Z.C., Bui T.A., Sonoda E., Takata M., Takeda S., Iliakis G. (2001). Efficient rejoining of radiation induced DNA-double strands breaks in vertebrate cells deficient in genes of the Rad52 epistatis group. Oncogene.

[Yannoneetal2001] Yannone S.M., Roy S., Chan D.W., Murphy M.B., Huang S., Campisi J., Chen D.J. (2001). Werner syndrome protein is regulated and phosphorylated by DNA-dependent protein kinase. J Biol Chem.

